# Adaptation and development trade-offs: fluvial sediment deposition and the sustainability of rice-cropping in An Giang Province, Mekong Delta

**DOI:** 10.1007/s10584-016-1684-3

**Published:** 2016-04-30

**Authors:** Alexander D. Chapman, Stephen E. Darby, Hoàng M. Hồng, Emma L. Tompkins, Tri P. D. Van

**Affiliations:** 1 Geography & Environment, University of Southampton, Southampton, SO17 1BJ UK; 2 Research Institute for Climate Change, Can Tho University, Can Tho, Vietnam; 3 College of Environment and Natural Resources, Can Tho University, Can Tho, Vietnam

**Keywords:** Adaptation, Sediment, Mekong Delta, Rice, Trade-off

## Abstract

Deltas around the globe are facing a multitude of intensifying environmental change and development-linked pressures. One key concern is the reduction in the quantity of suspended sediment reaching and building floodplains. Sediment deposition provides multiple services to deltaic social-ecological systems, in particular, countering the subsidence of the delta-body, and providing plentiful nutrients. Experiencing particularly rapid change is the Vietnamese Mekong Delta (VMD). In An Giang Province an increasing number of high dyke rings, which exclude the flood and facilitate triple rice-cropping, simultaneously prevent much of the sediment load from reaching the floodplain. This paper explores the trade-offs implicit in the decision to shift from (i) doublecropping (higher sediment deposition) to (ii) triple cropping (lower sediment deposition) by asking: what is the impact of the shift on VMD farmers? Is it sustainable? And what is the significance of the associated sediment exclusion? A novel survey of An Giang rice farmers was conducted, investigating key agricultural practices, and uniquely, the farmers’ estimates of annual sediment deposition depth. The survey elicits some key changes under the adapted system (ii), particularly, unsustainable trajectories in the yield to fertiliser ratio which penalise land-poor farmers. Furthermore, the value (to farmers) of the sediment contribution to agricultural fertilisation which is lost due to triple-cropping is estimated at USD 15 (±5) million annually. We argue that our growing understanding of the importance of sediment in the deltaic social-ecological system may be revealing an emergent risk; arising from conflicting long and short-term adaptation and agricultural development objectives.

**Electronic supplementary material:**

The online version of this article (doi:10.1007/s10584-016-1684-3) contains supplementary material, which is available to authorized users.

## Introduction

In deltas around the globe climate change-induced eustatic sea-level rise (Church et al. 2013), natural subsidence, and development-linked subsidence are outstripping the delta-building power of sediment accretion, causing deltas to ‘drown’ and thereby threatening the lives and livelihoods of hundreds of millions of people (Syvitski et al. 2009). The supply of fluvial sediment to deltas is known to be a critical factor in maintaining delta surfaces above rising sea levels. While it is understood that fluvial sediment loads are potentially significantly affected by climate change, the processes involved are currently poorly understood, making it very difficult to predict the future supply of fluvial sediment reaching the world’s deltas (Shrestha et al. 2013). Despite these uncertainties, there is broad consensus that, at the global scale, fluvial sediment loads are declining, primarily due to trapping as a result of the construction of dams upstream (Syvitski and Kettner 2011), a practice which looks set to continue throughout the 21st century (e.g. Fearnside and Pueyo 2012; Kondolf et al. 2014).

However, the quantity of sediment which reaches the floodplain and potentially contributes to delta building is not simply a function of the fluvial sediment flux (Manh et al. 2014). For example, in many of the world’s deltas endogenous structures and management practices are a key factor in controlling the flow dynamics and exchanges of sediment between rivers and their floodplains (Hung et al. 2014). Of particular significance are the extensive networks of canals and dykes that are commonly encountered in the world’s deltas, at densities up to 1.4 km/km^2^ (ibid). These canal and dyke networks are explicitly designed to protect crops from intense flooding events and to provide year-round irrigation. The compartments which are formed facilitate highly productive agriculture in deltas around the world, such as the Pearl (Seto et al. 2002), Nile (Nixon 2003), Mekong (Hung et al. 2014), Ebro (Ibáñez et al. 1997), and Skagit (Hood 2004). Yet, and in the context of likely declining fluvial sediment loads, such networks also disconnect floodplains from their rivers and potentially limit the supply of fluvial sediment reaching the surface of the delta.

Sediment deposition within these compartments is not only important for maintaining delta surfaces above rising sea levels. The nutrients that are bound to deposited sediments have made deltaic soils and ecosystems some of the most productive on the planet (Venterink et al. 2006). The continued provision of such natural nutrients can, therefore, reduce the need for costly chemical fertilisers (Nixon 2003). Farmers have been reaping the economic benefits of natural sediment deposition for centuries through practices such as digging sediment out of the canals and spreading it over the floodplain, as in the Nile Delta (ibid); engineering siltation projects, as in the Ebro Delta (Ibáñez et al. 2013); and making strategic decisions on dyke height which allow overflow, as was traditionally the case in the Mekong Delta (Manh et al. 2014). Local farmers and land managers must therefore make a trade-off between achieving either maximal sediment deposition to aid delta building and natural nutrient replenishment, or to promote flood prevention that limits sediment deposition. This trade-off has grown in significance in recent years as strategic sediment delivery for land-building has been identified as a key adaptation strategy to sea-level rise (Ibáñez et al. 2013).

While there is a wealth of research into plant nutrient uptake and management and issues of soil degradation and agricultural intensification (Tilman et al. 2002), very little work has gone into examining this trade-off from a socioeconomic perspective and assessing its wider implications for adaptation and development. In this paper we seek to address this gap through the use of a novel social survey approach that quantifies the socioeconomic trade-offs of the physical relationship between sediment-bound nutrient availability and rice yield in the Vietnamese Mekong Delta. The structure is as follows: the study area is introduced and the aim of the study is set out; the data collection methods are then outlined; the results are presented with specific regard to each of our four objectives; and finally, we conclude by discussing both the local policy implications and the broader significance of the study.

## Study area

**Fig. 1:**
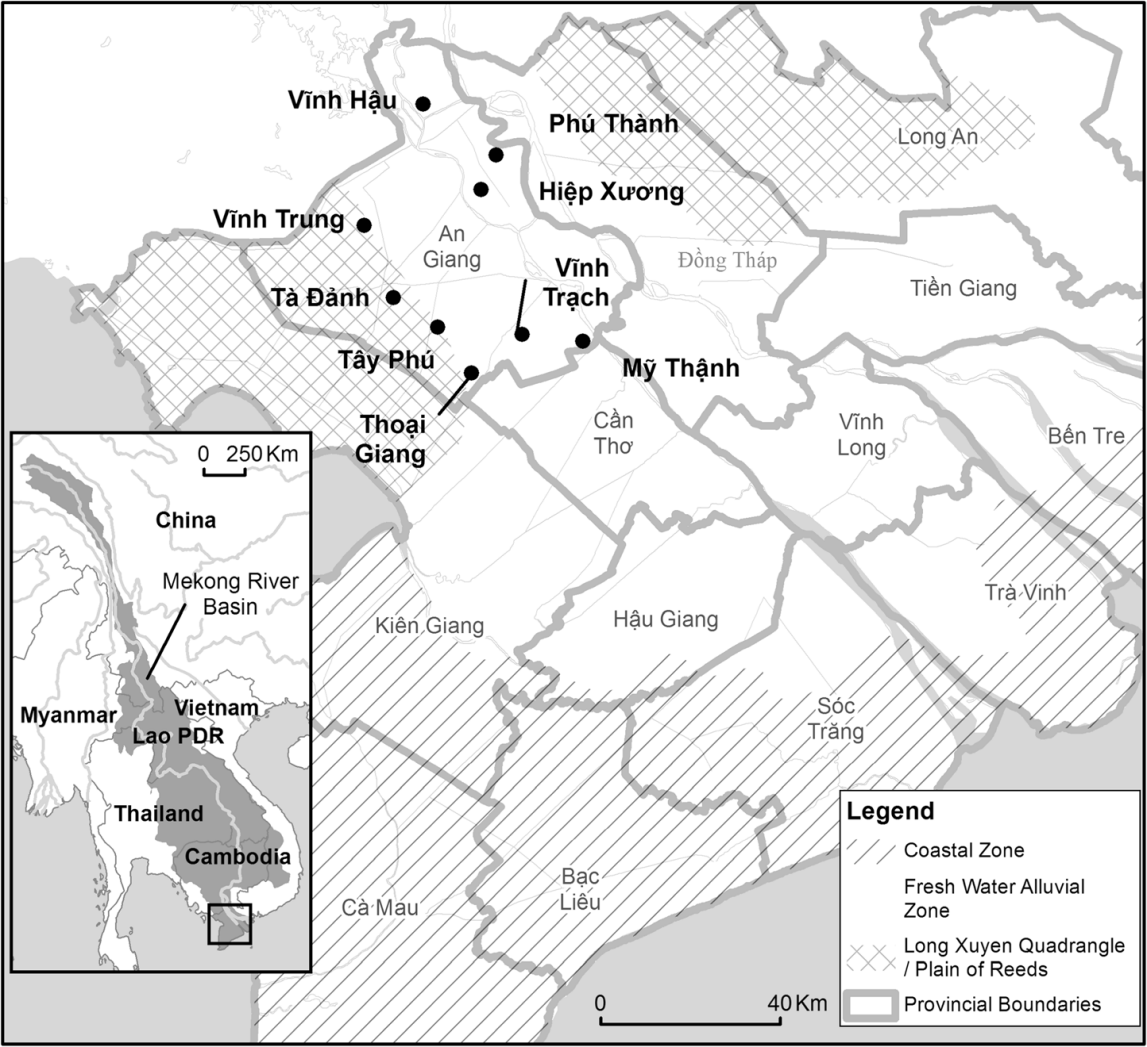
*Inset*: the Mekong River Basin and the location of the study site. *Right*, the locations of the communes visited in An Giang Province. *Highlighted*: the coastal zone, where saline intrusion dominates and land use comprises aquaculture, supplemented by fruit and wet season rice; the fresh water alluvial zone, where triple-rice cropping dominates; and the Long Xuyen Quadrangle and Plain of Reeds where multiple land uses operate and double rice-cropping is still practiced

Around 18 million people live and work in the Vietnamese Mekong Delta (VMD; Fig. 1), the ‘rice-bowl’ of South East Asia; but they are threatened by some of the most rapid and systemic environmental changes in the world (Smajgl et al. 2015). The Mekong River and its tributaries, which by modern standards were in almost ‘pristine’ ecological condition only two decades ago (Dudgeon 2011), now face (i) dam regulation on a large scale (Kuenzer et al. 2013), and (ii) highly uncertain hydrological regime changes as a consequence of anthropogenic climate change (Lauri et al. 2012). The former development (i) will result in large reductions (up to 96 %) in suspended sediment loads reaching the Delta due to trapping behind the proposed cascade of dams (Kondolf et al. 2014), and the latter (ii), will have further unknown impacts on the sediment supply (Shrestha et al. 2013). Both of these changes are, theoretically, significant for their impact on land-building rates in a region that is, on average, no more than 5 m above sea-level (Van et al. 2012); and where sea-level rise and ground-water extraction-induced subsidence are predicted to create 0.42–1.54 m of additional inundation hazard by 2050 (Erban et al. 2014). For these reasons, the Mekong Delta is one of the most at-risk regions on the planet (Oppenheimer et al. 2014).

The VMD’s network of river and canal dykes extends over ca. 180,000 km (Manh et al. 2014) and in many areas has been heightened from an average crest of 2.5 (low) to 4.5 (high) metres above sea-level (Hung et al. 2014). These changes are altering the timing, and substantially reducing the duration, of the fluvial inundation which brings sediment deposition (Manh et al. 2014). Since around 2006 such changes have been made in the name of climate change adaptation (Vietnamese Government 2011; MARD 2008). The stated aim of the “upgrade” to the dyke network in the Vietnamese Government’s “National Strategy on Climate change” (2011) is to: *“effectively cope with floods, droughts, seal level rising, and salt contamination in the context of climate change”*. In practise, however, there is an additional motive of agricultural development, as the dyke heightening facilitates a shift from double to triple rice-cropping which, at least in the short-term, increases total rice production and export without any change in planted area (GSO 2014). Indeed, encouraging the multiplication of rice crops has been a policy in the VMD since the early 1990’s (Vietnamese Government 1996). This system, in which climate change impacts, adaptation, and development objectives are interacting in such a complex manner is a system with susceptibility for ‘emergent risk’, a concept that has been explored in the IPCC’s 5th assessment report (Oppenheimer et al. 2014) and which is defined as: *“A risk that arises from the interaction of phenomena in a complex system”*.

The risks associated with triple rice-cropping, and the sustainability thereof, have been studied in some detail and, for example, yield and input efficiency declines are known to be worse than in the more traditional double-cropping systems (Dawe et al. 2000). Nevertheless, there is uncertainty as to the optimal choice between double and triple-cropping systems when the conflicting objectives (e.g. total rice production or long term livelihood sustainability) of different stakeholders are considered (Pham et al. 2004). Further study is required, but particularly in deltaic environments where the exclusion of fluvial sediment deposition is an additional impact of the two to three crop shift.

In the VMD, sediment deposition is known to especially provide Potassium (K), a staple fertiliser in rice agriculture (Hoa et al. 2006). Manh et al. (2014) estimate that the annual deposition of sediment-bound nutrients can supply over half of the fertilisation (N, P, K) needed for a season of rice agriculture. Under the old, low dyke, double-cropping system, these nutrients were typically deposited during the 2–3 months of fluvial inundation brought by the summer monsoon. In An Giang Province, the focus of this study and where over 85 % of the land in production is dedicated to rice (GSO 2014), these nutrients are potentially of significant economic value. One report has estimated, delta-wide, an annual loss of USD 24 million would be incurred by a 75 % reduction in nutrients reaching the Delta, calculated on the bulk-weight of suspended sediment-bound nutrients reaching the delta and at 2010 prices (ICEM 2010). However, this estimate ignored the impact of local paddy management practices of farmers. The cost-free input of sediment-bound nutrients is now under threat in the VMD as, at least in triple-cropping areas, the floodplain receives only a few days of inundation and deposition between crops, as facilitated by sluice gate operation (Manh et al. 2014).

In the northern VMD, some authors have already linked the switch to the new triple-cropping system, which is synonymous with the adaptation action described above, to negative impacts which share some of the traits of ‘maladaptation’ as described by Barnett and O’Neill (2010). Many of these alleged negative impacts, such as increasing the inequality between the landless and the land owners (Birkmann et al. 2012; Pham 2011), increasing the prevalence of pests and disease (Pham 2011), and reducing agricultural productivity (Garschagen et al. 2012) remain largely unquantified. Two further impacts were identified in the ‘Mekong Delta Plan’ (MDP 2013): flood water exclusion has the potential to exacerbate downstream flooding; and the associated exclusion of sediment accretion was recognised as counterproductive to sustaining the long-term integrity of the delta. The provincial government have some awareness of these problems and therefore recommend a 3-3-2 cropping cycle across An Giang province. In this 3-3-2 cropping cycle compartments are fully opened to allow flood inundation and sediment deposition once every 3 years. In the other 2 years, paddies are farmed intensively and require around 269 days of labour annually (Garschagen et al. 2012). Uptake of the 3-3-2 cycle is poor (Sakamoto et al. 2009).

### Aims and objectives

In answer to calls published in a number of high profile documents and papers (e.g. Manh et al. 2014; MDP 2013; Dobermann et al. 2004) herein we explore the rice production-sediment trade-off inherent in the double to triple cropping switch (and the 3-3-2 system)—with a particular focus on the role of sediment-bound nutrients.

Present knowledge encompasses only the physical processes involved in sediment deposition in the Northern provinces of the Mekong Delta (e.g. Manh et al. 2014; Hung et al. 2014). Using a household survey which focuses on An Giang Province this paper looks at the problem from a social perspective. We analyse agricultural trends to establish:

1. What is the impact of the double to triple rice-cropping shift on VMD farmers? And is it sustainable?
2. What is the significance of sediment loss during the shift?
3. Who does the shift benefit?
4. What are the implications for adaptation/emergent risk in the VMD?

In the process, an economic valuation of deposited sediment’s contribution to rice fertilisation is made which aims to contribute to a further gap in the knowledge required for systemic evaluation of upstream dam developments (Kuenzer et al. 2013; ICEM 2010).

## Methods

### Framework

Concepts from the DPSIR (*Drivers – Pressures – States – Impacts – Responses)* framework are used to structure our methods, analysis, and discussion. DPSIR is a simple framing tool, pioneered by the OECD (2003), for investigating complex issues of environmental change and/or degradation. The framework encourages the presentation of a problem’s cause and impacts in a format that is clear and easily translated into policy (Tscherning et al. 2012). Our utilisation of the framework follows on from that of Suckall et al. (2014) who also investigated adaptation/development trade-offs. Our focus is primarily on the evaluation of the trade-offs implicit in the *response* phase, (i.e. the changes made to the VMD dyke network) to *pressures* exerted by climate change and development *drivers. Responses* to climate change *pressures* may constitute ‘adaptations’ but, there is an increasing body of literature to suggest that such *responses* may have their own, second-order, *impacts*, especially, when *responses* attempt to tackle non climate-driven *pressures* simultaneously (Suckall et al. 2014).

### Data collection

#### Drivers, pressures, states, and impacts

Strategic decisions on the VMD’s hydraulic operations are guided by targets set at the national level, but specific decisions on the system’s management are made and controlled at the provincial level of governance. We conducted a semi-structured focus group with four high ranking provincial officials to clarify the context within which decisions on hydraulic operations are made. We aimed to establish the *drivers* of change they face, the *pressures* they are responding to, and the *responses* they have enacted. Discussion was facilitated with the participants around five key questions:

- What are the objectives which guide your actions?
- Can you rank those objectives?
- What are the threats you face in meeting those objectives?
- How are you responding to those threats?
- Have there been any impacts of those *responses*?

#### Reponses and second-order impacts

Our objective here was to examine the impacts of high dykes, triple-cropping, and sediment exclusion from a socioeconomic perspective. Floodplain sedimentation can be highly spatially and temporally variable and hence difficult to measure; data is sparsely available on local rates in the VMD (Manh et al. 2014). However, VMD farmers are aware of the fertilising effects of fluvial sediment, and most typically work any sediment left by inundation into an even spread around their paddy. While previous research has shown that perceptions may differ from physical measurements (Meze-Hausken 2004), the strong local knowledge of the phenomenon meant we posited that farmer perceptions could provide a meaningful estimate. Furthermore, our objective was to build into our analysis a direct link between sediment and livelihoods considerate of local behaviours and management practices (over which farmer perceptions are likely to have considerable influence). Data collection, including on physical processes, was therefore performed using a structured, quantitative survey among heads of rice-farming households.

Asking farmers to make quantitative estimates of the depth of the sediment (if any) left behind by the monsoon was a new approach to analysing the deltaic environment. To help this process farmers were presented with visual aids (a scale showing different depths, and a diagram). Some simple validation checks of the farmers’ reported sediment values and greater detail on the data collection and analysis can be found in the Supplementary information.

**Fig. 2:**
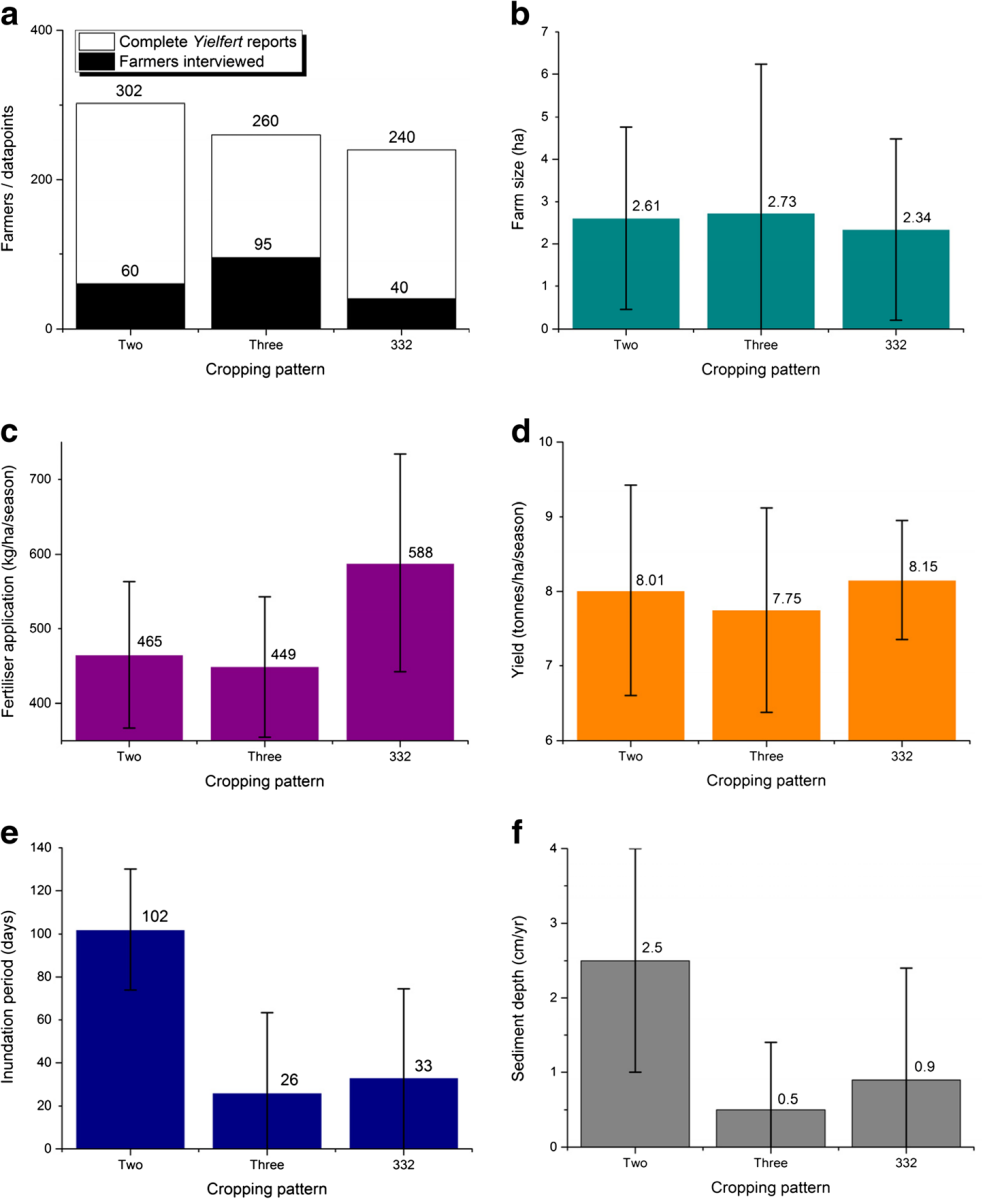
Graphs summarising the survey results in each cropping category. Graph **a** shows the number of farmers interviewed and the number of complete *Yielfert* values they supplied. Graph **b** shows the mean farm size and standard deviation. Graph **c** shows the mean seasonal fertiliser application and standard deviation. Graph **d** shows the mean yield and standard deviation. Graph **e** shows the mean period of inundation and standard deviation. Graph **f** shows the annual sediment deposition depth and standard deviation

The temporal and geographic locations of cropping patterns are changeable and difficult to map (Sakamoto et al. 2009). As a result, a random selection process was applied to the primary sampling units (PSU), the commune authorities, and a random walk technique was performed within the selected PSU to seek out a representative sample of rice farming households in the relatively homogenous environment of rice growing compartments. Following this sampling procedure, a total of 195 rice farmers were interviewed across nine communes of An Giang province (see Fig. 1). Figure 2a shows the cropping systems they operated. Interviews were conducted in April and May 2014 and were conducted by native-speaking enumerators and translators from Can Tho University.

### Data analysis

#### Regression model

Technical efficiency (TE) as first established by Farrell (1957) is widely regarded as a good indicator of the status and sustainability of an agricultural system (De Koeijer et al. 2002). In rice farming, the yield to fertiliser ratio, *Yielfert*, is the primary indicator of TE and its level and trend indicate the system’s status and the success of policies (Khai and Yabe 2011). However, we hypothesised that any free fertilisation contributed by sediment deposition would be detected in higher levels of *Yielfert*. To test this hypothesis a general linear regression model (GLM) was built using the data collected from the social surveys (Eq. 1). Independent variables were selected for the regression analysis which addressed the research objectives listed in Section 2.1. The key independent variable representing the efficiency gain farmers receive from the annual deposition of sediment on their floodplain was termed *β*_*3*_. Also included was farm size (*β*_*2*_ *)*, which was utilised as an indicator of the wealth of the farmer (targeting objective 3); and finally, fertiliser application (*β*_*1*_ *)* was included which, as a key determinant of *Yielfert,* allowed a basic check of the model’s trends against expectations (e.g. Witt et al. 1999). Specifically, the greater the fertiliser applied the higher the farmer operates on the production function and hence, the lower the *Yielfert* that would be expected. Additional independent variables were initially included which may have been pertinent (e.g. farm distance from river or canal, and the period of paddy inundation) but these (not statistically significant) variables were dropped during the model refinement process, thereby improving the fit of the model. Analysis was performed with variables *β*_1_–*β*_3_ nested within the different cropping systems (i.e. two/three/3-3-2).

1 $$ Yielfert={\beta}_{1\left( kg/ season/ ha\right)}(fertiliser)+{\beta}_{2(ha)}\left( farm\; size\right)+{\beta}_{3\left( cm/ yr\right)}\left( sediment\; depth\right) $$

#### Calculating the economic value of sediment

Once modelled, the influence of sediment (*β*_3_) on *Yielfert* could be translated into economic terms by converting its relative influence on *Yielfert* into its relative influence on the yield to fertiliser price ratio. This value could then be extrapolated to the operations of all of the farmers within each cropping system (Eq. 2) across the province.

2 $$ {Y}_{\left(VND/ yr\right)} = {f}_{\left( kg/ season/ ha\right)}.{c}_{\left(VND/ kg\right)}.{\beta}_{3\left(VND/VND/ cm\right)}.{s}_{\left( cm/ yr\right)}.{v}_{(seasons)}.{a}_{(ha)} $$

*Y*: provincial value of sediment
*f*: fertiliser applied
*c*: average cost of fertiliser
*β*_3_: cost efficiency gain per centimetre of sediment
*s*: average depth of sediment
*v*: crops per year
*a*: area in production

## Results and discussion

### Drivers, pressures, and the response

As discussed in Section 2, there is evidence that the heightening and lengthening of the VMD dyke network began before climate change adaptation became a formal policy objective. Policy documents make it clear that the dyke network’s development was aimed at stabilising the environment for the safety of residents and crops, and to allow multiplication of crops (Vietnamese Government 1996). However, while the senior decision makers attending the focus group agreed that the network’s historical objectives were aimed at improving yields, their discussion clearly highlighted that climate change adaptation is central to the network’s current objectives. Participants cited climate change as the *driver* behind three of their five most important *pressures*, respectively: (1) the growing threat to people and (2) the growing threat to crops from floods intensified by climate change and (3) the growing threat to crops from saline intrusion exacerbated by sea-level rise (currently only present in the western regions of An Giang). The other two *pressures* related to development *drivers*, they were: (4) the growing demand for irrigation to sustain livelihoods, especially during the dry season, and (5) the challenge of supplying water for the domestic use of a growing population. The participants highlighted the high dyke rings constructed, such as one completed around Phú Tân District in 2007, as their *response* to the *impacts (*e.g. the high floods of 2000 and 2001, see Birkmann et al. 2012) of the above *pressures*.

Finally, the participants were asked about any *state* changes and second-order *impacts* that have resulted from their *response*. The participants emphasised the success these initiatives have had in reducing the numbers of flood deaths in An Giang Province. But, the participants also recognised that there were issues related to the sediment excluded by the third rice-crop now being grown. Participants explained that they are encouraging farmers to implement the 3-3-2 cropping cycle to increase sediment deposition, but also highlighted the role of sediment as a poorly understood area requiring further research. With regard to enforcing the 3-3-2 cycle, one participant observed: “*we cannot tell the farmers to reduce their production because the farmers need the production to sustain their livelihoods*”.

### What is the new *state* of the An Giang agricultural system? And is it sustainable?

**Fig. 3:**
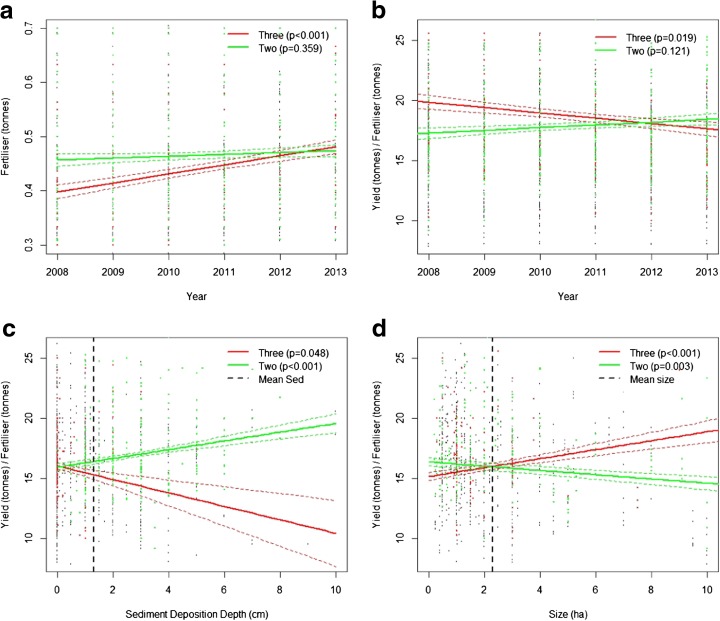
Regression lines (GLM) modelling the differences between the three and two-crop categories. *P*-values are labelled on each graph and standard errors are represented by *dashed lines*. The data points corresponding to the modelled cropping category are highlighted in their corresponding *colour*. Graph **a** models fertiliser over time. **b** models the *Yielfert* ratio over time. **c** models the *Yielfert* ratio against sediment deposition depth. **d** models the *Yielfert* ratio against farm size

The key indicators of yield, fertiliser application, and *Yielfert* (TE), when analysed as time-independent, seasonal, per-hectare, values, showed no significant difference between the triple (high dyke, i.e. adapted) and double (low dyke, i.e. unadapted) cropping patterns (Fig. 2). However, the temporal trends between cropping patterns are notably different (Fig. 3b). Other studies (e.g. Diep 2013) would suggest a positive temporal trend in *Yielfert* should be expected as agricultural practices, seed varieties, and the quality of inputs improve. But, a strongly-significant (*p* = 0.019) negative *Yielfert* trend was reported in the triple-cropping areas, in contrast to the expected positive trend (albeit with *p* = 0.121) which was found in the double-cropping paddies (Fig. 3b). The unexpected negative trend in the triple-cropped areas may be interpreted as being driven primarily by increasing rates of fertiliser application (Fig. 3a and Table 1) which suggests farmers are seeking to compensate for declining productivity, and in the long term the practice may not be sustainable (e.g. Garschagen et al. 2012). By 2013, triple-cropping farmers were applying more fertiliser per crop than their double-cropping counterparts, meaning annual application has increased disproportionately between patterns.^1^ The importance of such a trend grows when trends in global fertiliser prices are taken into account, which, though subject to considerable temporal variability, have been increasing rapidly (World Bank 2014). Triple-cropping farmers will be more susceptible to fertiliser price spikes and furthermore, increasing seasonal fertiliser application means an increasing workload for farmers.

**Table 1:** Summary of the *Yielfert * regression model’s characteristics

Model characteristics: *R* ^*2*^ *= 0.639 F = 104.9*_*11, 1185*_ *p < 0.001*					
Variable	Province	Cropping system	Estimate	SE	*p*-value
Intercept	n/a	n/a	12.306	0.263	<0.001
Fertiliser application (*β*_1_)	An Giang	Two	−0.012	<0.001	<0.001
		Three	−0.017	<0.001	<0.001
		332	−0.010	<0.001	<0.001
Farm size (*β*_2_)	An Giang	Two	−0.070	0.024	0.003
		Three	0.148	0.043	<0.001
		332	0.072	0.036	0.048
Sediment depth (*β*_3_)	An Giang	Two	0.145	0.041	<0.001
		Three	−0.222	0.113	0.048
		*332*	*−0.051*	*0.048*	*0.290*

Variables significant to *P* > 0.05 are highlighted in italics

### Impacts of the response

The depth of sediment perceived by the farmers was in the order of five times greater in the double-cropping areas than the triple (Fig. 2f). The influence of the farmer estimated sediment depths was highly statistically significant in its influence on *Yielfert* (Fig. 3c). Double-cropping farmers reported on average around two and a half centimetres of sediment. The regression coefficient, *β*_3_, = 0.145 (±0.041) indicates this deposition improved their average annual input efficiency by around 0.3 tonnes of yield per tonne of fertiliser (Table 1). This equates to an approximate 2 % improvement in agricultural efficiency and applies across all crops grown in the year (intra-annual variation cannot be detected in this analysis). This gain appears minor when compared with Manh et al.’s (2014) estimate—that sufficient nutrients are contained within sediment deposits to meet half of a season’s fertilisation needs. But, VMD farmers operate very fine margins, and this gain would be worth approximately USD 190 (±50) annually to the average farmer (at 2014 prices this represents 9 % of GDP per capita; World Bank 2014). The vast majority of triple-cropping farmers reported no, or negligible, deposition depth, the average was around half a centimetre (in keeping with Hung et al. 2014 who estimated around 0.6 cm in the neighbouring province of Dong Thap). On occasion the triple-cropping farmers did report notable deposition levels but, in conversation, they frequently associated it with a minor dyke breach that had also damaged their crop. Thus, in the triple-cropping system, sediment reduced farmers’ input efficiencies (*β*_3_ = −0.222, *p* = 0.048, Fig. 3c).

#### The economic value of sediment

A figure was calculated using the method detailed in Section 3.2.2 for the maximum potential value of sediment if all paddies were operating the double cropping system and receiving the average deposition depth of 2.5 cm/year. For a *β*_*3*_ = 0.145 (±0.041) tonnes(yield)/tonne(fertiliser)/cm the value of sediment-bound deposited nutrients to An Giang paddy rice farmers would be in the order of USD 26 (±9) million, as of May 2015 (details of the assumptions made in calculating this figure can be found in the Supplementary information). This figure is of a similar order of magnitude to that of ICEM, which was estimated in 2010, and costed for 75 % sediment reduction across the entire delta. However, the value of the sediment currently being utilised by the minority two-crop farmers was USD 11 (±4) million meaning USD 15 (±5) million of potential value (fertiliser savings) is being lost annually by the high dykes’ exclusion of sediment in An Giang. A key drawback of these estimates, and a reason to treat them with caution, is that no research has yet reliably estimated the destination/s of sediment being excluded by the high dykes—downstream low-dyke farmers may be receiving more sediment due to upstream exclusion.

#### Who does the shift benefit?

The loss of this free nutrient input will have implications for the local farmers. Particularly, it may penalise the land-poor who operate the finest profit margins. Using farm size as a proxy for wealth, a notable difference was observed in *Yielfert*’s response to varying wealth levels between cropping systems (Table 1). Under the triple-cropping system *Yielfert* improved as farm size increased (Fig. 3d). Such a finding is in line with most other studies (e.g. Khai and Yabe 2011). Input efficiency (*Yielfert*) often improves with farm size as it is linked with factors such as higher farmer education levels, access to advanced equipment, and better soil quality (ibid). Such factors mean that fewer inputs are wasted (particularly fertiliser and seed) due to bad practice and inefficient distribution.

However, notably, under the two-crop system, the expected relationship could not be detected. Double-cropping gave no input-efficiency advantage to land wealthy farmers, indeed there was a significant (*p* = 0.003) negative trend (Fig. 3d). Explanations can only be hypothesised, but, for land-poor double-cropping farmers the burden of applying fertiliser is currently lower on three counts: (i) the total annual application is lower, (ii) by 2013, the per-season application was lower, and furthermore, (iii) the effects of free sediment-bound fertilisation are wealth-independent. All three of these factors will reduce the signal in the model of the advantages held by richer farmers, possibly allowing other factors to dominate, such as the ease of managing a smaller plot of land. These, and indeed other disadvantages, may or may not also apply to farmers who do not own the land they manage. But, as only 1.4 % of the farmers interviewed for this study rented their land, investigation of this factor was not possible.

### Second-order *responses*

**Fig. 4:**
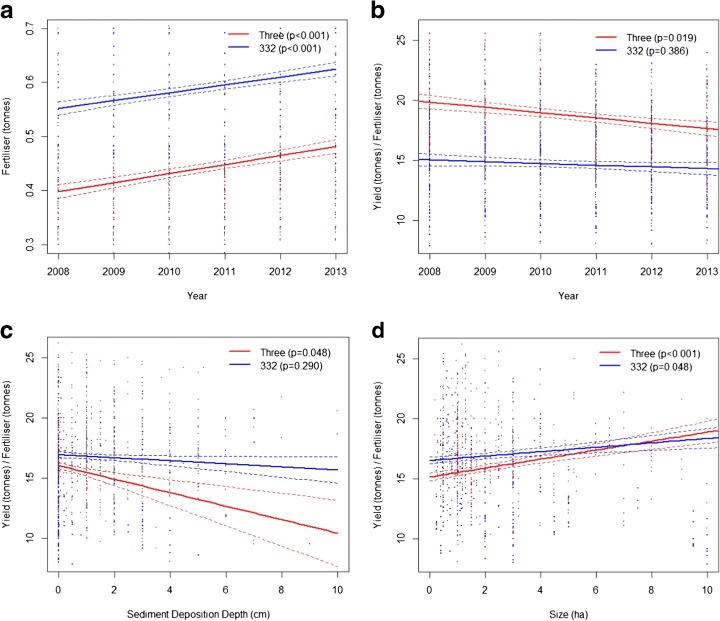
Regression lines (GLM) modelling the differences between the 3 and 332 cropping categories. *P*-values are labelled on each graph and standard errors are represented by *dashed lines*. The data points corresponding to the modelled cropping category are highlighted in their corresponding *colour*. Graph **a** models fertiliser over time. **b** models the *Yielfert* ratio over time. **c** models the *Yielfert* ratio against sediment deposition depth. **d** models the *Yielfert* ratio against farm size

One official response to the impacts described above has been implemented, the 3-3-2 cropping cycle (others, such as described in footnote 1, are being trialled). Of the nine PSUs visited, two were operating this system—implemented by the district level of governance. Figure 4 summarises the performance of the 3-3-2 cropping cycle against the triple-cropping system. While 3-3-2 farmers appear to be operating a more input intensive system, the trajectories of change they report in their survey responses are not statistically distinguishable from those in the triple-cropping system. Further investigation into this response, and potentially others, is required. The benefits of operating the double-cropping system within the high dykes and facilitating inundation through sluice gate operation needs exploration. Some have suggested that the nature of sediment deposition (quantity and particle characteristics) is different when inundation results from sluice gate operation rather than dyke overflow due to the position, capacity, and physical barrier imposed by the gate (Hung et al. 2014). Furthermore, if triple-cropping is to continue, strategies for reducing its disproportionate impact on the poor may need exploration, such as fertiliser subsidies, price guarantees, and off-season income diversification assistance.

### What are the implications for adaptation and emergent risk in the VMD?

Issues of soil erosion/degradation in intensive agricultural systems are not new; nor is the finding that triple rice-cropping leads to declining productivity and input efficiency (e.g. Dawe et al. 2000). However, the concurrent exclusion of significant fluvial sediment deposition is a factor unique to the fluvial floodplain environment and hence absent from most otherwise comparable studies and indeed delta management plans. Encouraging sediment deposition with the aim of strategic land building is, however only a recently identified adaptation strategy (e.g. Ibáñez et al. 2013). Our study is the first to assess the role of sediment in underpinning deltaic agriculture from the farmers’ socioeconomic perspective. As such, newly identified risks have been associated with its exclusion through the use of high dykes. More broadly, issues such as the exacerbation of the land-rich vs land-poor divide, the declining fertility, sustainability, and profit margins in rice agriculture, and implicit second-order impacts such as the increased level of specialisation and greater workload necessitated by the shift might be regarded as maladaptive traits of the dyke heightening.

Commentators suggest that the control Vietnamese farmers have over their operations and local infrastructure is restricted by administrative structures at the national and provincial levels (Giesecke et al. 2013). As such, responsibility for ensuring the efficacy of adaptation lies, at least in part, with the decision makers we consulted. Our participants appeared firm in their position that triple-cropping is essential to livelihood success in the region (though they recognised the need for further research). The new evidence of maladaptive traits identified herein will now need balancing against the short-term flood protection service being provided by the dykes. It might be argued that the issues presented have been exacerbated by conflicting objectives of climate change adaptation and agricultural development in land-management plans. Indeed, the negative impacts documented herein may represent an emergent risk to communities in the VMD. Decision makers now face path dependency in An Giang, as such they will need supporting through further research and evaluation into practical policies (second-order adaptations, Birkmann 2011) such as the 3-3-2 cropping system and others (e.g. a return to double-cropping or diversification).

## Conclusions

The Mekong Delta’s farmers are attempting to sustain their livelihoods in a context of rapid environmental change, development, and a pressing need to adapt. This paper has looked at some key trajectories in the northern province of An Giang and has highlighted how an action stated to be adaptive is reshaping the socioeconomic system. This is the first study to have detected through quantitative means the influence of sediment upon that socioeconomic system. Our survey identifies some key trends associated with high dykes: the declining productivity of agriculture, the loss of free sediment-bound nutrients and their contribution to agricultural productivity and profitability (potentially worth USD 26 (±9) million), and the exacerbation of the divide between land-rich and land-poor farmers. These findings contribute to the wider debate on the future of the VMD (MDP 2013), they largely support proposals that sediment deposition should be strategically encouraged (Manh et al. 2014; Hung et al. 2014), but highlight that the benefits of such a policy lie not only in land building, but also in avoiding the maladaptive traits described above. This paper’s contribution to the wider academic community is the addition of evidence to a sparse body on how actions simultaneously targeting adaptation and development wins can interact to result in undesirable dynamics with potential for emergent risk.

## Electronic supplementary material

Below is the link to the electronic supplementary material.

ESM 1 (DOCX 1046 kb)

## Footnotes

1 There has already been some recognition of this issue (see Garschagen et al. 2012) and it is being targeted by projects such as the ‘Vietnam Low Carbon Rice Project’, see: http://dfat.gov.au/about-us/publications/Pages/vietnam-low-carbon-rice-project-design.aspx.
