# Supplementary material for: Adaptation and development trade-offs: fluvial sediment deposition and the sustainability of rice-cropping in An Giang Province, Mekong Delta
Source: Clim Change. 2016 Apr 30;137(3):593–608. doi: 10.1007/s10584-016-1684-3 (PMC7175674; doi:10.1007/s10584-016-1684-3)
Supplement: Supplementary file 1 — (DOCX 1046 kb) [file 10584_2016_1684_MOESM1_ESM.docx]

**This document contains supplementary information to the paper titled:**

**“Adaptation and Development Trade-offs: Fluvial sediment deposition and the sustainability of rice-cropping in An Giang Province, Mekong Delta”**

*Foreword:*

- *The original survey was conducted in both An Giang and Soc Trang provinces. 434 farmers were interviewed. However, the social-ecological system differs between the two provinces and, as the focus of this paper is on comparative analysis between cropping-systems and only the triple rice-cropping system was found in Soc Trang, comparisons were not possible and the decision was taken to remove Soc Trang from the analysis.*
- *In order to protect the anonymity of our focus group participants we are not able to go into any further detail on their background.*

**6. Extended Methods**

### 6.1 The survey

The actual number of surveys collected in each commune was determined by a number of practical aspects of data collection in the region including, the time spent with the local commune authorities, the time taken to access farmer households, and weather conditions. **Table 6.1** summarises the households visited. The survey was conducted using a random walk methodology within the Primary Sampling Unit (PSU – the commune) adapted from the WHO’s EPI cluster survey (Oliphant et al., 2006). The survey was designed to contain the minimal amount of “sensitive” information possible, and focus on easily quantifiable topics, in order to reduce potential biases in the data. As is the norm in Vietnam, representatives of the local government were present at interviews thus qualitative, subjective, questions about local policy (e.g. central dyke and sluice gate operations or rice growing targets) may have placed farmers in an uncomfortable position.

In order to identify any differences between PSUs which might hinder comparison, semi-structured interviews were conducted with each PSU’s administrative leader. These interviews sought out issues such as: localised pest and disease outbreaks, unusual rice varieties, and different agricultural technologies. No major issues were identified in An Giang province (in Soc Trang use of specialist, incomparable, rice varieties was found).

For the general survey, respondents were required to be directly responsible for the management of a rice paddy to be considered for interview. Respondents were first asked for basic farm characteristics such as size, ownership status, and number of workers. Second, they were asked to report the number of crops they grew, the fertiliser they applied, the yield they achieved, and the period of floodplain inundation allowed in each year between 2008-2013 (inclusive). In the case of yield and fertiliser, respondents were able to either supply their annual average or individual, seasonal, values. Respondents were permitted to provide estimates in any units, and were encouraged only to give answers they had confidence in. As such, respondents often provided less than the maximum six responses in each category (see Figure IIA). Thirdly, a similar process was performed asking for the respondent’s perception of the depth of sediment deposited by fluvial inundation in each of the last six years. Finally, a subset (n=30) of farmers were asked for the prices paid for fertiliser, and received for rice, in 2013 in order to corroborate secondary data that was collected from the provincial statistical yearbook (AGSO, 2013), the FAO (2014), and the World Bank (2014) which facilitated the economic calculations detailed below.

### 6.2 The results analysis

Statistical analysis was conducted in the R software package. Generalised Linear Models (GLMs) were utilised, as a widely accepted method of investigating the factors explaining the supply of an ecosystem service (Mouchet et al., 2014) and measuring agricultural trends over time (e.g. Dawe et al., 2000).

| **Table 6.1:** The number of farmers interviewed in each cropping category. | | | | |
| --- | --- | --- | --- | --- |
| Status | Crop Category | Code | Farmers interviewed | Communes |
| Post-adaptation | Three crops per year | *Three* | 74 | Vĩnh Trung, Tà Đảnh, Tây Phú, Vĩnh Trạch, Thoại Giang |
|  | Adapted three year cycle “3-3-2” | *332* | 40 | Hiệp Xương, Phú Thành |
| Under adaptation | Changed system during period | *Three* | 21 | Tà Đảnh, Mỹ Thậnh, Vĩnh Trung |
| Pre-adaptation | Two crops per year | *Two* | 60 | Mỹ Thậnh, Vĩnh Hậu, Vĩnh Trung, Tà Đảnh |
|  |  | **Total:** | 195 |  |

### 6.3 Is the survey methodology utilised reliable?

This study relied on farmers’ records and memories of recent practices. In similar situations information reliant on participant memory has proven to be highly accurate and detailed (Brondizio and Moran, 2008). For example, other studies within the region have successfully utilised household surveys to investigate shifts in farming systems over time (e.g. Bosma et al., 2006) and some have gone considerably further back into participant’s pasts (e.g. Polimeni et al., 2014). For the majority of participants in this survey rice growing was a vocation and their main (usually only) source of income, the skills for which have been passed between generations. A small number (unrecorded) of the visited households practiced aquaculture as a second source of income. As such, most participants knew their farming practices well and were capable of noting year to year shifts. The challenge of teasing out accurate data laid with the survey enumerators. In this case the enumerators had considerable experience in the execution of surveys in the rural parts of the Delta (a minimum of six previously completed) and were also postgraduate (or greater) students of agriculture and the environment at Can Tho University, located in the centre of the Delta. Nevertheless, training was provided to reinforce the core principles of the survey.

A particular feature this survey was the decision to ask the farmers to estimate a physical process: the depth of the sediment left behind on their paddy as the flood water subsided; and the length of the flood inundation they experienced. Farmers provided six responses, one for each year 2008-2013, the most common responses were 0 cm, 1 cm, and 2 cm, the maximum reported was 10 cm and the minimum 0 cm, hence, all of the responses might be regarded as within the ‘realms of possibility’.

Two basic logical tests and one validation test against secondary data were performed against the farmer’s reported sediment depths (centimetres) and inundation period (days) in order to validate the farmer’s observations. Those tests were conducted on the subset of farmers still influenced by the natural regime (i.e. the 2 crop farmers, n=60):

**Logical tests:**

- Can variance in the perceived sediment deposition depth be explained by a positive relationship with the perceived period of inundation? (expected answer: yes): yes, strongly 0.015 (±0.001) cm/day (p <0.001)
- Hence, can variance in the perceived sediment deposition depth be explained by a negative relationship with the height of the dykes? (expected answer: yes): yes, strongly, the presence of high dykes reduced deposition by 1.35 (±0.07) cm (p <0.001)

**Secondary data validation**

- Can variance in the perceived period of inundation be explained by a positive relationship with the MRC’s historical data on the number of days during which the water level breached 2.5m at Tan Chau? (expected answer: yes) : yes, weakly, 0.002 (±0.001) days/day (p =0.066)

### 6.4 Calculating the economic value of sediment

**Tables 6.2** and **6.3** provide more information on the process of calculating an economic value of sediment using the calculation presented in **Section 3.3.2.**

| **Table 6.2**: summary of the supplementary data used in the calculation of the economic value of sediment | | | |
| --- | --- | --- | --- |
| Variable | Survey estimate | Secondary data estimate (source) | Utilised |
| Fertiliser price | 11,700 (VND/Kg) | 8,200 (World Bank, 2014) | Survey |
| Rice price | 4,650 (VND/Kg) | 3,200 (FAO, 2014) | Survey |
| Area in 2 crop production | 64,997 (ha) | 84,259 (AGSO, 2013) | Secondary |
| Area in 3 crop production | 135,755 (ha) | 116,493 (AGSO, 2013) | Secondary |

| **Table 6.3**: Summary of steps in calculating the economic value of sediment | | | | | | | | |
| --- | --- | --- | --- | --- | --- | --- | --- | --- |
| Crops | *v* | β_3_ | SE | *f.c* | *a* | Seasonal value (‘000 VND) | Annual value (USD^1^) | SE (±USD) |
| 3 | 0.43 | -0.283 | 0.087 | 5,148 | 116,493 | -72,978,219 | *-10,045,178* | 3,088,094 |
| 2 | 2.53 | 0.104 | 0.036 | 5,452 | 84,259 | 120,872,083 | 11,091,726 | 3,839,444 |
| Value lost | 2.53 | 0.104 | 0.036 | 5,452 | 116,493 | 167,112,731 | 15,334,960 | 5,308,256 |
|  |  |  |  | **Total:** | 200,752 | **Total potential value:** | **26,426,686** | **9,147,699** |
| ^1^exchange rate correct as of 27/05/2015 at 1 (USD) : 21,795 (VND) | | | | | | | | |

### 6.5 References

AGSO. (2013). Statistical Yearbook of An Giang: 2012. Long Xuyen: An Giang Statistical Office.

Bosma, R. H., Phong, L. T., Kaymak, U., Berg, J. Van Den, Udo, H. M. J., Mensvoort, M. E. F. Van, & Tri, L. Q. (2006). Assessing and modelling farmers’ decision-making on integrating aquaculture into agriculture in the Mekong Delta. {NJAS} - Wageningen Journal of Life Sciences, 53(3–4), 281–300.

Brondizio, E. S., & Moran, E. F. (2008). Human dimensions of climate change: the vulnerability of small farmers in the Amazon. Philosophical Transactions of the Royal Society of London. Series B, Biological Sciences, 363(1498), 1803–9.

Dawe, D., Dobermann, a, Moya, P., Abdulrachman, S., Singh, B., Lal, P., … Zhen, Q.-X. (2000). How widespread are yield declines in long-term rice experiments in Asia? Field Crops Research, 66(2), 175–193.

FAO. (2014). An Giang Rice Price Data. Retrieved November 17, 2014, from http://www.fao.org/giews/pricetool/

Mouchet, M. a., Lamarque, P., Martín-López, B., Crouzat, E., Gos, P., Byczek, C., & Lavorel, S. (2014). An interdisciplinary methodological guide for quantifying associations between ecosystem services. Global Environmental Change, 28, 298–308.

Oliphant, N., Mason, J., Eisele, T., & Conkle, J. (2006). Sampling issues. New Orleans.

Polimeni, J. M., Iorgulescu, R. I., & Chandrasekara, R. (2014). Trans-border public health vulnerability and hydroelectric projects: The case of Yali Falls Dam. Ecological Economics, 98, 81–89.

World Bank. (2014). Overview of Commodity Markets. Retrieved November 17, 2014, from http://www.worldbank.org/en/research/commodity-markets

### 6.6 Additional figures

Figure 7.1: A different take on Figures IIIC. This time the data has not been separated by cropping pattern but into years in which two crops were grown and years in which three crops were grown regardless of cropping pattern (i.e. the 332 system has been included). A GLM regression line has been plotted for *Yielfert* against sediment and *95%* confidence intervals have been drawn instead of SE.


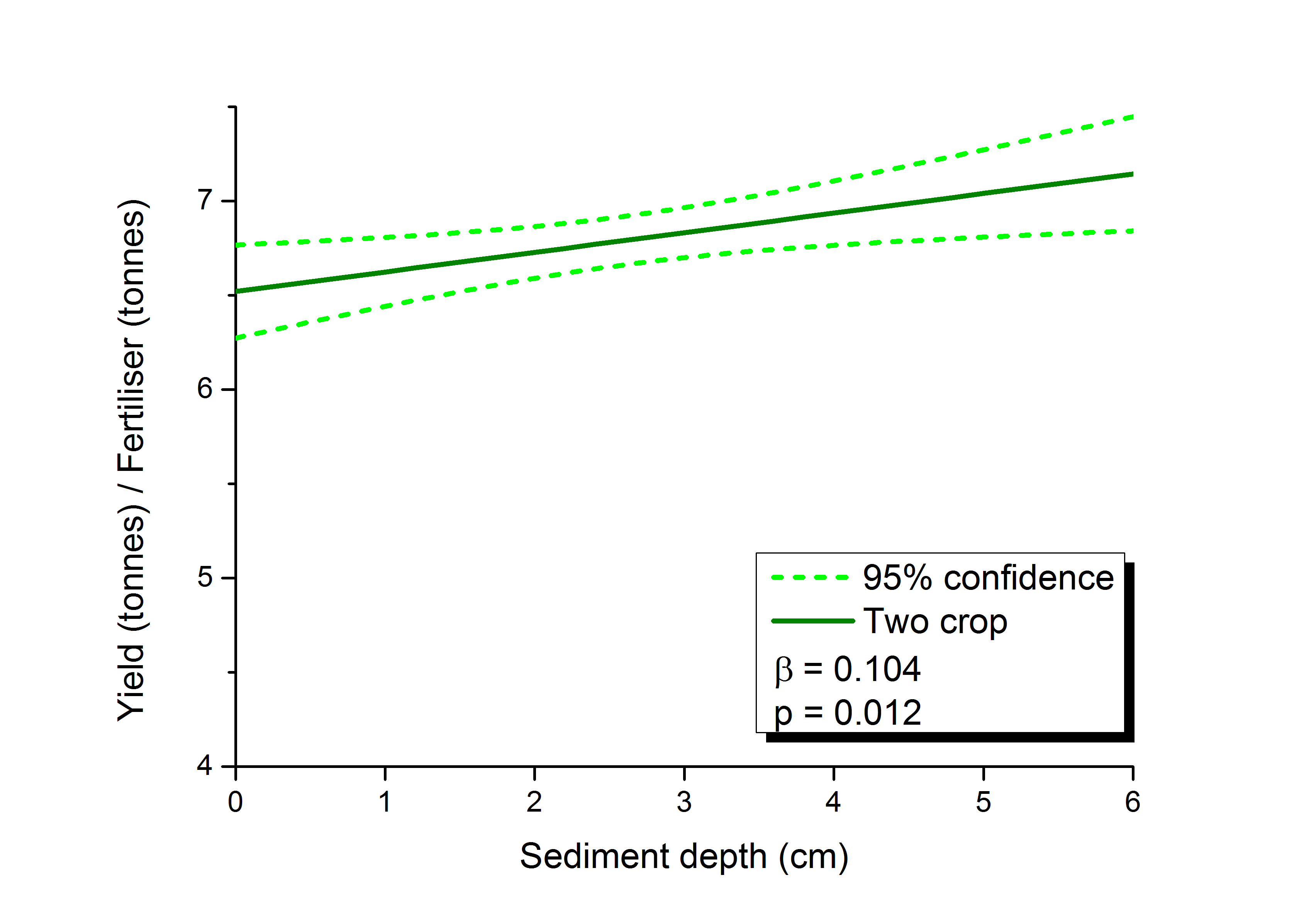

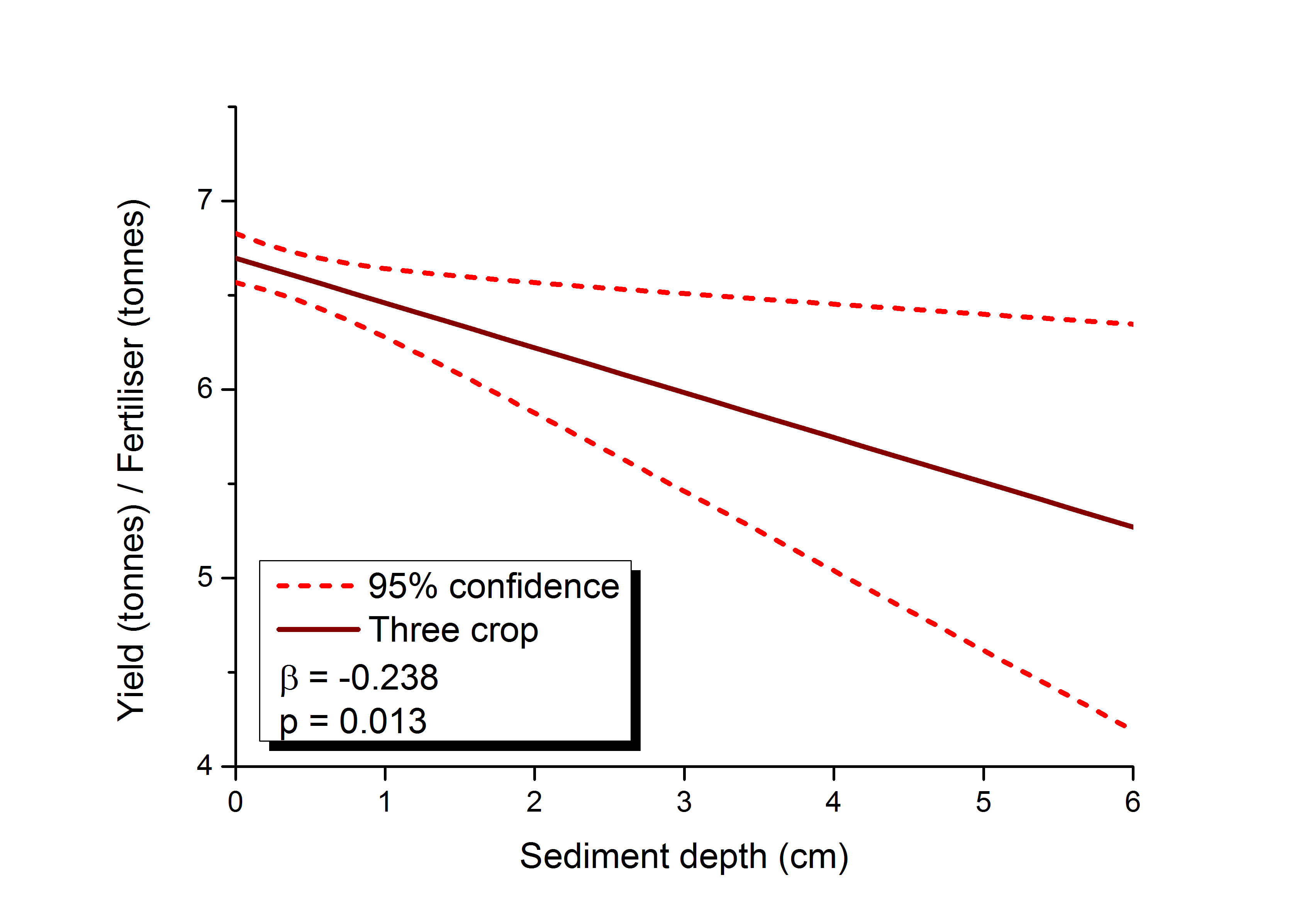


Figure 7.2: Mean daily suspended sediment concentration (SSC; black line) and the height of water (blue line) entering the VMD at Tan Chau (2005-2010) (data accessed through the MRC data portal and provided by SIWRR, Vietnam). Highlighted is the period during which farmers growing three crops tended to open sluice gates and manually flood their paddies.


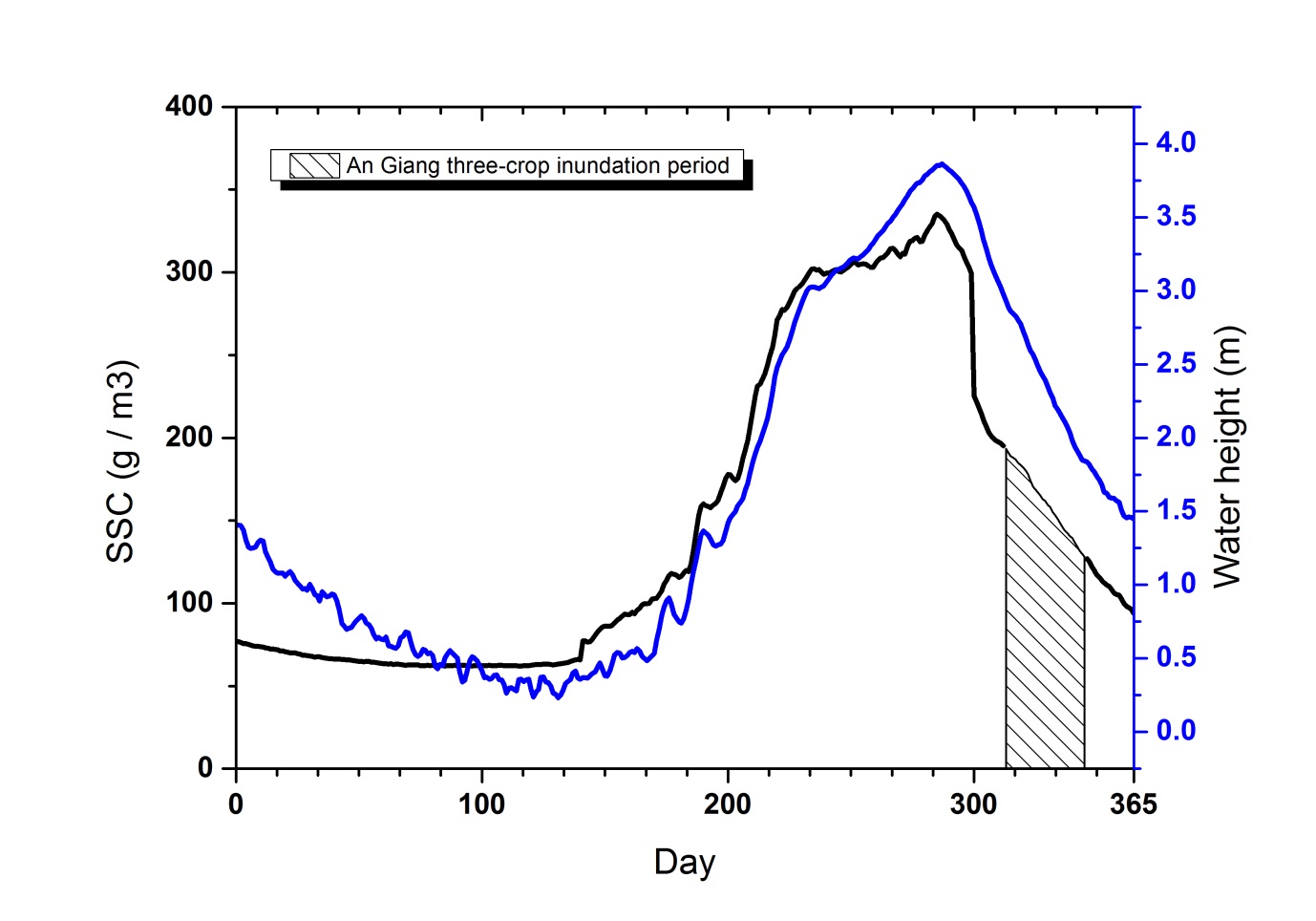


Fertiliser contribution

Net fertilisation

Time

Time

Net fertilisation

Figure 7.3: A conceptualisation of the increasing burden on fertiliser application during sediment loss.

Two-crop system

Three-crop system

Sediment contribution


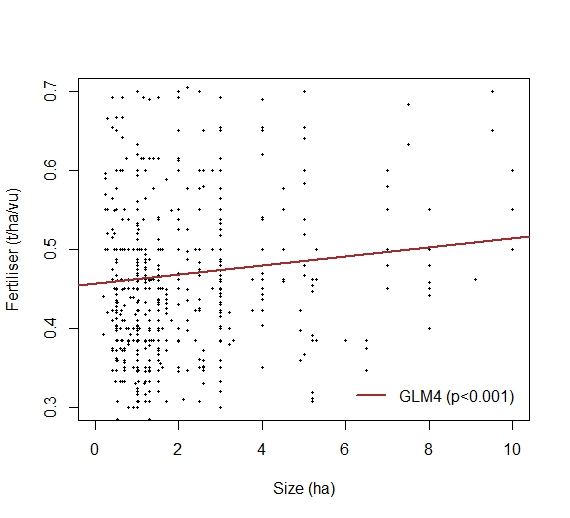


Figure 7.4: The significant relationship between fertiliser application and farm size across the whole dataset
